# Identification of a Novel RAMA/RON3 Rhoptry Protein Complex in *Plasmodium falciparum* Merozoites

**DOI:** 10.3389/fcimb.2020.605367

**Published:** 2021-01-18

**Authors:** Daisuke Ito, Jun-Hu Chen, Eizo Takashima, Tomoyuki Hasegawa, Hitoshi Otsuki, Satoru Takeo, Amporn Thongkukiatkul, Eun-Taek Han, Takafumi Tsuboi

**Affiliations:** ^1^Division of Malaria Research, Proteo-Science Center, Ehime University, Matsuyama, Japan; ^2^Division of Medical Zoology, Department of Microbiology and Immunology, Faculty of Medicine, Tottori University, Yonago, Japan; ^3^National Institute of Parasitic Diseases, Chinese Center for Disease Control and Prevention, Shanghai, China; ^4^Division of Tropical Diseases and Parasitology, Department of Infectious Diseases, Faculty of Medicine, Kyorin University, Mitaka, Japan; ^5^Department of Biology, Faculty of Science, Burapha University, Chonburi, Thailand; ^6^Department of Medical Environmental Biology and Tropical Medicine, Kangwon National University School of Medicine, Chuncheon, South Korea

**Keywords:** *Plasmodium falciparum*, merozoite, monoclonal antibody, rhoptry, rhoptry-associated membrane antigen, rhoptry neck protein 3

## Abstract

Malaria causes a half a million deaths annually. The parasite intraerythrocytic lifecycle in the human bloodstream is the major cause of morbidity and mortality. Apical organelles of merozoite stage parasites are involved in the invasion of erythrocytes. A limited number of apical organellar proteins have been identified and characterized for their roles during erythrocyte invasion or subsequent intraerythrocytic parasite development. To expand the repertoire of identified apical organellar proteins we generated a panel of monoclonal antibodies against *Plasmodium falciparum* schizont-rich parasites and screened the antibodies using immunofluorescence assays. Out of 164 hybridoma lines, 12 clones produced monoclonal antibodies yielding punctate immunofluorescence staining patterns in individual merozoites in late schizonts, suggesting recognition of merozoite apical organelles. Five of the monoclonal antibodies were used to immuno-affinity purify their target antigens and these antigens were identified by liquid chromatography-tandem mass spectrometry (LC-MS/MS). Two known apical organelle protein complexes were identified, the high-molecular mass rhoptry protein complex (PfRhopH1/Clags, PfRhopH2, and PfRhopH3) and the low-molecular mass rhoptry protein complex (rhoptry-associated proteins complex, PfRAP1, and PfRAP2). A novel complex was additionally identified by immunoprecipitation, composed of rhoptry-associated membrane antigen (PfRAMA) and rhoptry neck protein 3 (PfRON3) of *P. falciparum*. We further identified a region spanning amino acids Q_221_-E_481_ within the PfRAMA that may associate with PfRON3 in immature schizonts. Further investigation will be required as to whether PfRAMA and PfRON3 interact directly or indirectly.

## Introduction

Malaria causes approximately a half a million deaths annually, mainly *via* infection with *Plasmodium falciparum* (WHO, 2019). To initiate intraerythrocytic development in humans, *P. falciparum* merozoites invade erythrocytes. Merozoite apical organelles—rhoptries, micronemes, exonemes, and dense granules—have been studied for their role in erythrocyte invasion. Before invasion some organelle components are discharged on the surface of merozoite. Once the merozoite recognizes and forms a tight junction between the erythrocyte membrane and its apical pole, the apical organelles discharge their protein contents into the moving junction and developing parasitophorous vacuole (PV). The apical organelles disappear after merozoite internalization within an erythrocyte, suggesting transient roles of their molecular contents during merozoite invasion (Cowman and Crabb, 2006). The apical organelles have thereby inspired analysis of the biological and immunological characteristics of their component proteins, as well as their candidacies for vaccine and drug development (Preiser et al., 2000; Kats et al., 2006; Kaneko, 2007).

Numerous rhoptry bulb proteins have been identified, including the high-molecular weight (HMW) proteins that form a complex consisting of PfRhopH1/Clag, PfRhopH2, and PfRhopH3 (Campbell et al., 1984; Holder et al., 1985; Lustigman et al., 1988; Sam-Yellowe et al., 1998; Kaneko et al., 2001; Kaneko et al., 2005); and the low-molecular weight complex (LMW) proteins consisting of PfRAP1, PfRAP2, and PfRAP3 (Ridley et al., 1990; Saul et al., 1992; Baldi et al., 2002). These protein complexes have been implicated in erythrocyte invasion (Siddiqui et al., 1987; Cooper et al., 1988; Harnyuttanakorn et al., 1992) and channel-mediated nutrient uptake (Counihan et al., 2017; Ito et al., 2017; Sherling et al., 2017). Another rhoptry bulb protein, rhoptry-associated membrane antigen (PfRAMA), is involved in rhoptry biogenesis, the merozoite invasion process, formation of the PV, and interacts with both PfRAP1 and PfRhopH3 (Topolska et al., 2004; Richard et al., 2009). The proteins were identified using monoclonal antibodies generated against parasite extracts (Campbell et al., 1984; Ridley et al., 1990; Saul et al., 1992; Doury et al., 1994; Sam-Yellowe et al., 2001) or proteomic analyses of purified merozoite rhoptries (Sam-Yellowe et al., 2004; Sanders et al., 2005; Gilson et al., 2006; Sanders et al., 2007). In addition to the rhoptry bulb proteins, the merozoite rhoptry neck proteins PfRON2, PfRON4, and PfRON5 form a moving junction complex together with a micronemal protein, PfAMA1, in *P. falciparum* (Collins et al., 2009; Richard et al., 2010). Therefore, the PfRON2/PfAMA1 complex proteins are highlighted as novel asexual blood-stage malaria vaccine candidates (Srinivasan et al., 2014).

A limited number of merozoite apical organellar proteins in micronemes, rhoptries, exonemes, and dense granules have been extensively assessed for their role in erythrocyte invasion and growth (Counihan et al., 2013; Cowman et al., 2017). The identification of novel merozoite apical organellar proteins is essential for the cumulative understanding of erythrocyte invasion, and therefore we attempted to expand the repertoire of apical organellar proteins and their partner molecules. In this study we have generated monoclonal antibodies (mAbs) against *P. falciparum* schizont-rich antigens that recognize the apical region of merozoites. We report here the immunofluorescence assay-based characterization of 12 newly obtained mAbs which react with apical organelles, and the identification of immunoaffinity-purified target antigens by liquid chromatography-tandem mass spectrometry (LC-MS/MS) analysis. We additionally describe the identification and validation of a novel PfRAMA/PfRON3 rhoptry protein complex of *P. falciparum*.

## Materials and Methods

### Parasite Culture

*P. falciparum* NF54 strain asexual stage parasites were maintained in continuous culture of human erythrocytes (blood group O^+^) obtained from the Japanese Red Cross Society, essentially as described (Ito et al., 2013; Morita et al., 2017).

### Fractionation of Schizont-Rich Parasites and Soluble Antigen Preparation

To obtain parasite specimens, mature schizonts were enriched to 65%–75% parasitemia *via* 65% Percoll-sorbitol centrifugation (Dluzewski et al., 1984). The pellets were treated with tetanolysin (3 µg/ml, Biological Laboratories, Campbell, CA) to remove hemoglobin without loss of parasite proteins present in the PV space as described (Hiller et al., 2003; Lopez-Estrano et al., 2003), and washed with phosphate-buffered saline (PBS) containing cOmplete protease inhibitor cocktail (Roche, Mannheim, Germany). Schizont-rich parasites (~10^8^) were disrupted by sonication (10 s pulse, 30 s rest, repeated 10 times) on ice in PBS supplemented with cOmplete protease inhibitor cocktail. Undisrupted cells and debris were removed by centrifugation at 21,600 × *g* for 15 min at 4°C. The resulting supernatant fractions were stored at −80°C and subsequently used as soluble antigen for mouse immunization, enzyme-linked immunosorbent assays (ELISA), and western blot analyses.

### Monoclonal Antibody Production

Mouse monoclonal antibodies (mAbs) were produced at Kitayama Labes (Ina, Japan). Briefly, three BALB/c mice (female) 8-weeks old were immunized in their foot pads with 50 µg of soluble antigen of *P. falciparum* mature schizonts, formulated with Freund’s complete adjuvant for the first immunization and with Freund’s incomplete adjuvant 2 weeks later. Six weeks after the second immunization an intravenous boost with the same amount of soluble fraction in PBS was administered, and lymphocytes from the inguinal lymph nodes were used to fuse with P3-X63-Ag8-U1 myeloma cells to produce hybridoma cells. Culture supernatants from hybridomas were initially screened for reactivity against immunogen by ELISA and secondarily with indirect immunofluorescence assays (IFA) using mature *P. falciparum* schizonts as antigen. Positive hybridoma cells were cloned by two rounds of limiting dilution and the antibody isotypes were determined using a monoclonal antibody isotyping kit (Santa Cruz Biotechnology, Santa Cruz, CA). Cloned cell lines were expanded as ascites in mice primed with Pristane (Wako, Osaka, Japan), and immunoglobulin G (IgG) was purified from ascitic fluid using a MAbTrap kit (GE Healthcare, Camarillo, CA).

### Immunofluorescence Assays

Thin smears of schizont-rich *P. falciparum*-infected erythrocytes were prepared and stored at −80°C. The smears were thawed, fixed with 4% paraformaldehyde at room temperature for 10 min, permeabilized with PBS containing 0.1% Triton X-100 at room temperature for 15 min, and blocked with PBS containing 5% non-fat dry milk at 37°C for 30 min. The smears were then incubated with both mouse monoclonal antibodies and rabbit polyclonal antibodies as counter staining at 37°C for 1 h, followed by incubation at 37°C for 30 min with both Alexa Fluor 488-conjugated goat anti-mouse IgG and Alexa Fluor 546-conjugated goat anti-rabbit IgG (Invitrogen, Carlsbad, CA) as secondary antibodies (1:500). Nuclei were stained with 4′,6-diamidino-2-phenylindole (2 μg/ml, DAPI). Slides were mounted in ProLong Gold Antifade (Invitrogen) and viewed under a 63× oil-immersion lens. High-resolution image capture and processing was performed using a confocal scanning laser microscope (LSM5 PASCAL or LSM710; Carl Zeiss MicroImaging, Thornwood, NY). Images were processed in Adobe Photoshop (Adobe Systems, San José, CA).

### Immunoelectron Microscopy

Parasites were fixed and embedded in LR White resin (Polysciences, Warrington, PA) and ultrathin sections were immunostained as described (Ito et al., 2011). Samples were examined with a transmission electron microscope (JEM-1230, JEOL, Tokyo, Japan).

### SDS-PAGE and Western Blot Analysis

Parasite soluble antigens were extracted in SDS-PAGE loading buffer, incubated at 4°C for 6 h, and subjected to electrophoresis under non-reducing and reducing conditions on 12.5% polyacrylamide gels (ATTO, Tokyo, Japan). Proteins were then transferred to 0.2 μm PVDF membranes (GE Healthcare). The proteins were immunostained with antibodies followed by horseradish peroxidase conjugated secondary antibody (GE Healthcare) and visualized with Immobilon Western Chemiluminescent HRP Substrate (Millipore, Billerica, MA) on a LAS 4000 Mini luminescent-image analyzer (GE Healthcare). The relative molecular masses of the proteins were estimated with reference to Precision Plus Protein Standards (BioRad, Hercules, CA).

### Affinity Purification of Target Proteins and Identification by Liquid Chromatography-Tandem Mass Spectrometry

Preparations of enriched late *P. falciparum* parasite schizonts were lysed for 1 h in extraction buffer [50 mM Tris–HCl, 0.2 M NaCl, 5 mM EDTA, 0.2% Nonidet P-40 (NP40; Nacalai Tesque, Kyoto, Japan), pH 7.4, containing 1 μg/ml leupeptin, 1 μg/ml pepstatin A, and 1 mM 4-(2-aminoethyl)-benzenesulfonyl fluoride hydrochloride (Wako)]. The lysate was centrifuged at 15,000 × *g* for 10 min at 4°C, and then target proteins were purified from the parasite lysate by affinity chromatography using a monoclonal antibody-conjugated Formyl-Cellulofine (Seikagaku-Kogyo, Japan) column as described (Kaneko et al., 2001). The following experiments were conducted at APRO SCIENCE (Naruto, Japan). Briefly, the purified protein was resolved by 10% SDS-PAGE under reducing conditions, and the expected individual target bands were excised from the gels. The extracted protein from each band was then digested overnight with trypsin (Thermo Fisher Scientific), and the resulting peptide fragments were fractionated by reverse phase high-performance liquid chromatography (EASY-nLC 1200, Thermo Fisher Scientific) and analyzed on a Q Exactive Plus mass spectrometer (Thermo Fisher Scientific). The obtained peptide mass fingerprints were used to search a *P. falciparum* protein sequence database (PlasmoDB, http://plasmodb.org) using the MASCOT program (Perkins et al., 1999).

### Production of Recombinant PfRAMA Proteins and Antisera

The *pframa* (PF3D7_0707300) nucleotide sequence of the strain 3D7 was obtained from PlasmoDB. To generate specific antibodies, three regions of *pframa* were amplified and expressed as recombinant proteins using the wheat germ cell-free protein synthesis system (WGCFS, CellFree Sciences, Matsuyama, Japan) as described (Tsuboi et al., 2008). Briefly, the constructs included full-length PfRAMA (PfRAMA_FL) excluding the signal peptide and GPI-anchor signal sequences (encompassing 768 aa, D_32_ to I_799_), the N-terminal region of PfRAMA (PfRAMA_N, encompassing 214 aa, D_32_ to D_245_), and the C-terminal region of PfRAMA (PfRAMA_p60, encompassing 277 aa, K_482_ to F_758_). Target regions were PCR amplified from *P. falciparum* NF54 blood-stage cDNA using sense primers with an XhoI restriction site and antisense primers with a BamHI site (in lowercase letters in the primer sequences below); specifically, PfRAMA-sense (5’-ctcgagGATCATAATATTAAGAATAATAATTGTATTA-3’), PfRAMA_FL-antisense (5’-ggatccCTATTTACTTATCAATTGTTTCTCTTCCTTA-3’), PfRAMA_N-antisense (5’-ggatccCTAATCGTCGTAATCATATTCTTCGCT-3’), PfRAMA_p60-sense (5’- ctcgagAAAAAAATGGTCTTTTATGATTTATAC-3’), and PfRAMA_p60-antisense (5’- ggatccCTAGAAAATTTTATTATTATTTTCTAATAATGT-3’). The amplified fragments were then restricted and ligated into the WGCFS vector pEU-E01-G(TEV)-N2 to fuse a GST-tag and TEV recognition site at the N-terminus of the target sequences (CellFree Sciences). The recombinant GST-PfRAMA proteins were captured using a glutathione-Sepharose 4B column (GE Healthcare), and the recombinant proteins were eluted by on-column cleavage with 60 U of AcTEV protease (Invitrogen). The detailed methods are described (Ito et al., 2011). To generate antisera against each recombinant PfRAMA protein, immunization was performed at Kitayama Labes (Ina, Japan). Briefly, two female BALB/c mice were immunized subcutaneously with 20 μg of purified PfRAMA with Freund’s adjuvant. A Japanese white rabbit was also immunized subcutaneously with 250 μg of purified PfRAMA with Freund’s adjuvant. All immunizations were performed 3 times at 3-week intervals, and then antisera were collected 2 weeks after the third immunization. We used additional mouse and rabbit polyclonal antibodies: anti-PfAMA1 (PF3D7_1133400), Q_25_-K_546_; anti-PfRON3_2 (PF3D7_1252100), D_1686_-K_1884_; and anti-PfRAP1 (PF3D7_1410400), M_1_-D_782_ that were generated and validated previously (Ito et al., 2011).

### Immunoprecipitation

Immunoprecipitation was carried out as described (Ito et al., 2011). Briefly, proteins were extracted from late schizont pellets in PBS with 1% Triton X-100 containing cOmplete protease inhibitor cocktail. After centrifugation the supernatants (50 µl) were preincubated at 4°C for 1 h with 40 µl of 50% protein G-conjugated beads (GammaBind Plus Sepharose, GE Healthcare) in NETT buffer (50 mM Tris–HCl, 0.15 M NaCl, 1 mM EDTA, and 0.5% Triton X-100) supplemented with 0.5% BSA (fraction V, Sigma-Aldrich). Aliquots of recovered supernatants were incubated with purified IgG from rabbit polyclonal antibody, and then 40 µl of a 50% protein G-conjugated bead suspension was added. After 1 h incubation at 4°C, the beads were washed once with NETT–0.5% BSA, once with NETT, once with high-salt NETT (0.5 M NaCl), once with NETT, and once with low-salt NETT (0.05 M NaCl and 0.17% Triton X-100). Finally, proteins were eluted from the protein G-conjugated beads with 0.1 M glycine–HCl (pH 2.5), and then immediately neutralized with 1 M Tris pH 9.0. The supernatants were used for western blot analysis using mouse antibodies.

## Results

### Monoclonal Antibody Production and Apical Organelle Recognition by Immunofluorescence Assays

**Figure 1:**
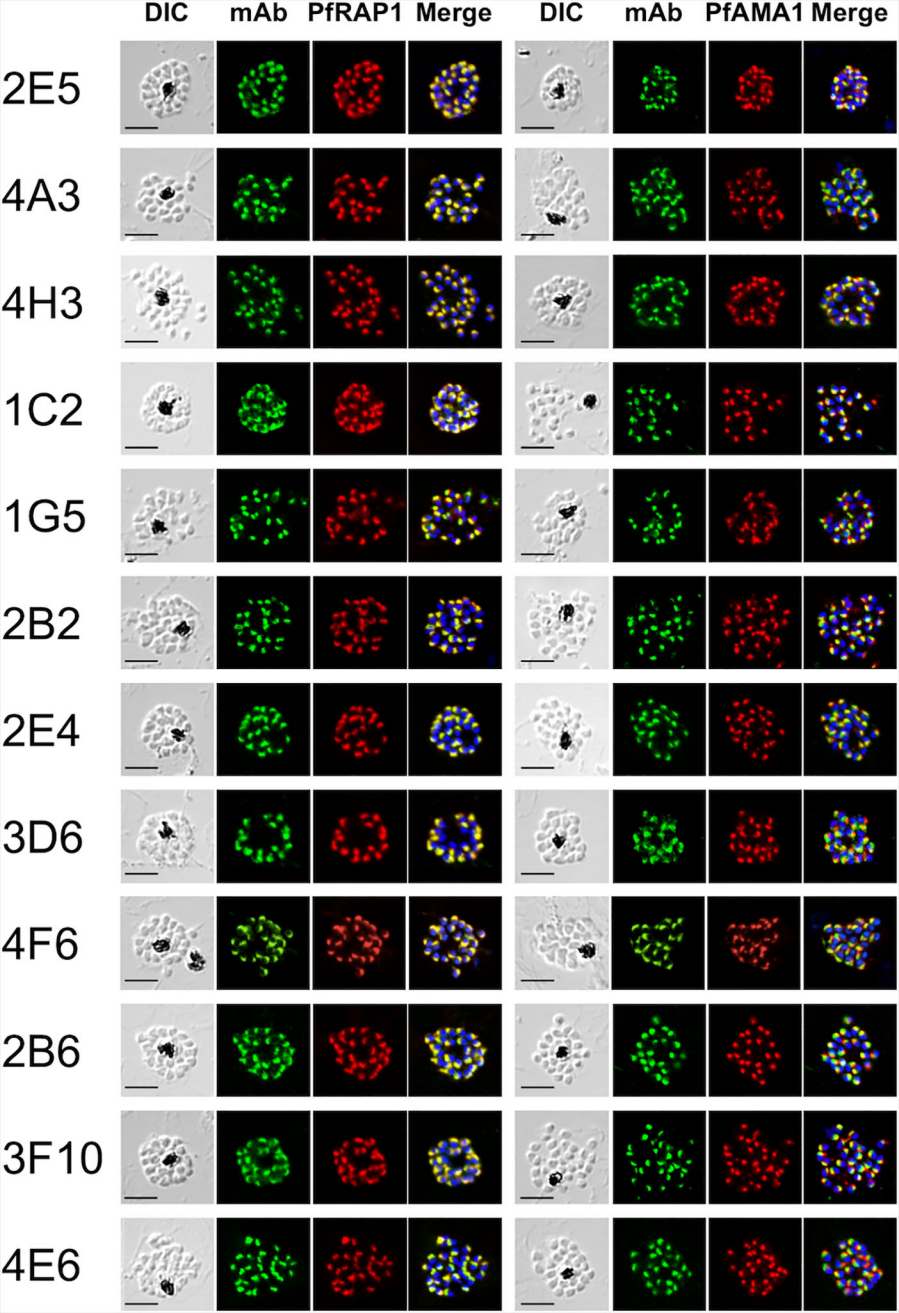
Monoclonal antibodies react with the apical end of *P. falciparum* merozoites. Mature schizont stage parasites were dual-labeled with each mouse mAb and rabbit antisera against either PfRAP1 (rhoptry body marker) or PfAMA1 (microneme marker). Nuclei were visualized with DAPI in the merged images shown in the right most panels. DIC, differential interference contrast microscopy. Merge, created by merging the IFA and nuclear-staining images. A representative image out of at least three independent experiments is shown for each mAb. Bars represent 5 μm.

Out of the 164 ELISA positive mAbs obtained against immunogens, only 12 (~7%) reacted by IFA against late schizont parasites with a punctate staining pattern suggestive of recognition of merozoite apical organelles (**Figure 1**). To predict target organelles, dual labeling IFA was performed with PfRAP1 as a rhoptry bulb marker and PfAMA1 as a microneme marker. All 12 selected mAbs colocalized with PfRAP1 but not with PfAMA1, suggesting recognition of the merozoite rhoptry bulb (**Figure 1**).

### Monoclonal Antibodies Recognized Distinct Parasite Antigens by Western Blot Analysis

To classify target antigens recognized by the 12 mAbs, we first determined the mAb isotype from the culture supernatant and then each mAb was purified from mouse ascitic fluid using a MAbTrap kit (GE Healthcare). We were unable to obtain purified mAbs from two clones, 2B2 and 3D6 (**Supplementary Figure 1**). Western blot analysis of schizont-rich parasite lysates was then performed with purified mAb to confirm reactivity and to predict the molecular weights of the target parasite native antigens. The mAb clone 4A3 (IgG1 isotype) reacted with antigens of approximately 60 and 52 kDa size; 4H3 (IgG1) with 100 and 95 kDa antigens; 1C2 (IgG1) with a 150 kDa antigen; 1G5 (IgG2a) with 170, 60, 45, 40, and 30 kDa antigens; 4F6 (IgG1) with a 47 kDa antigen; 2B6 (IgG1) with a 60 kDa antigen; 3F10 (IgG1) with 100 and 52 kDa antigens; and 4E6 (IgG1) with 60 and 50 kDa antigens. The mAb clones 2E5 (IgG1) and 2E4 (IgG1) did not react with parasite antigens under this condition. Overall, eight western blot-positive mAbs recognized distinct parasite antigens (**Supplementary Figure 1**).

### Target Antigen Identification by Liquid Chromatography-Tandem Mass Spectrometry From Immunoaffinity-Purified Parasite Proteins

We successfully obtained 5 mAb clones from mouse ascites in sufficient quantity to generate immunoaffinity columns. To identify target proteins recognized by the mAbs, schizont-rich parasite extracts were immunoaffinity-purified by affinity columns conjugated with each mAb followed by LC-MS/MS analysis. The mAbs were categorized based on whether they recognized LMW or HMW rhoptry protein complexes, or other proteins.

### Monoclonal Antibodies 4F6 and 4H3 Recognize the Low-Molecular Weight PfRAP Complex Proteins

**Figure 2:**
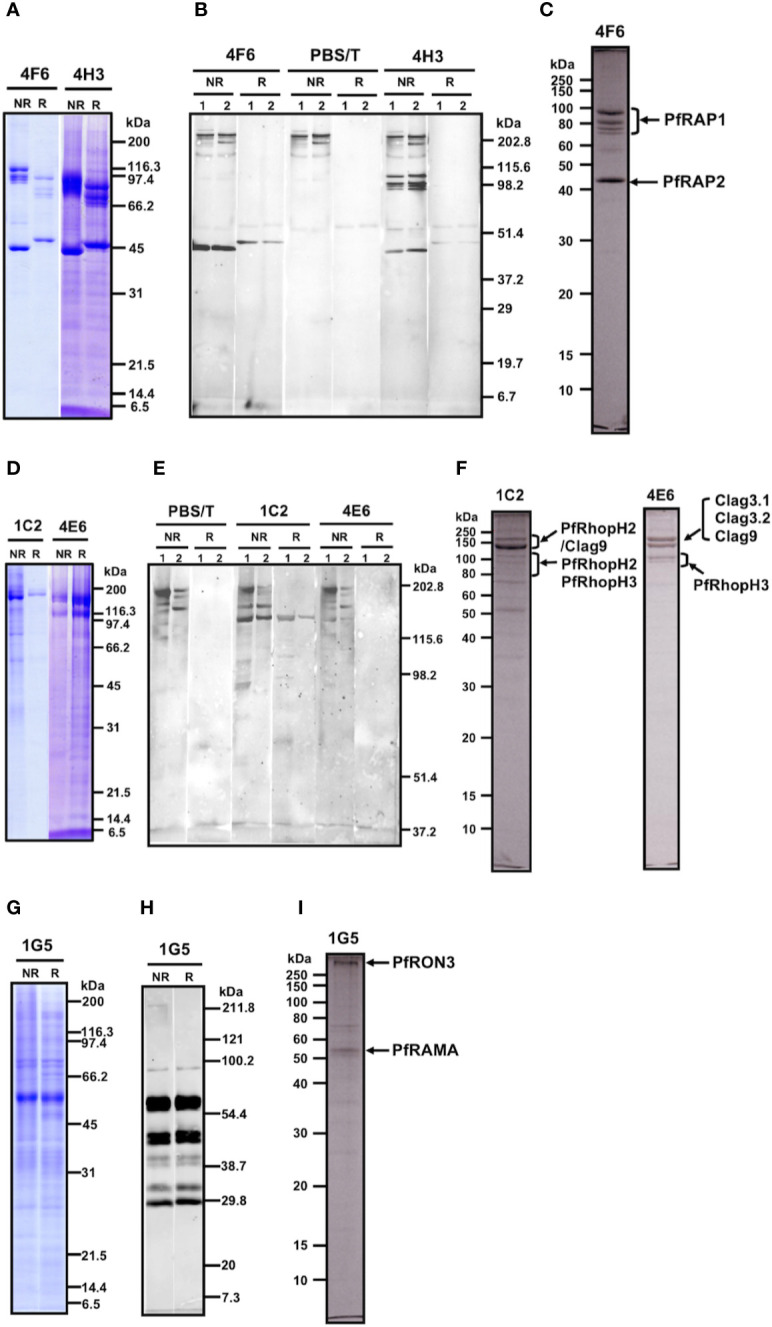
**(A–C)** Analyses of the immunoaffinity-purified proteins using affinity columns conjugated with either mAb 4F6 or 4H3. **(A)** The elution fractions were resolved by 12.5% SDS-PAGE under reducing or non-reducing conditions. **(B)** Western blot analyses of elution fractions. Elution fractions from 4F6 (lane 1) and 4H3 (lane 2) affinity columns were resolved by 12.5% SDS-PAGE and the proteins were probed with either mAb 4F6 or 4H3. PBS/T serves as a negative control staining. **(C)** Protein bands used for the LC-MS/MS analyses. The elution fraction from the 4F6 column was resolved by 10% SDS-PAGE under reducing conditions and the target bands (arrows) were excised from the gel. Proteins identified by LC-MS/MS are indicated. **(D–F)** Analyses of the immunoaffinity-purified proteins using affinity columns conjugated with either mAb 1C2 or 4E6. **(D)** The elution fractions were resolved by 12.5% SDS-PAGE under reducing or non-reducing conditions. **(E)** Western blot analyses of elution fractions. The elution fractions from the 1C2 (lane 1) and 4E6 (lane 2) affinity columns were resolved by 7.5% SDS-PAGE and the proteins were probed with either mAb 1C2 or 4E6. PBS/T serves as a negative control staining. **(F)** Protein bands used for the LC-MS/MS analyses. The elution fractions from the 1C2 and 4E6 columns were resolved by 10% SDS-PAGE under reducing conditions and the target bands (arrows) were excised from the gel. Proteins identified by LC-MS/MS are indicated. **(G–I)**. Analyses of the immunoaffinity-purified proteins using an affinity column conjugated with mAb 1G5. **(G)** The elution fraction was resolved by 12.5% SDS-PAGE under reducing or non-reducing conditions. **(H)** Western blot analysis of elution fraction. Elution fractions from the 1G5 affinity column were resolved by 12.5% SDS-PAGE and the proteins were probed with mAb 1G5. **(I)** Protein bands used for the LC-MS/MS analyses. The elution fraction from the 1G5 column was resolved by 10% SDS-PAGE under reducing conditions and the target bands (arrows) were excised from the gel. Proteins identified by LC-MS/MS are indicated.

Western blot analysis of parasite lysates indicated that mAb 4F6 recognized a distinct antigen from that recognized by 4H3 (**Supplementary Figure 1**), but the SDS-PAGE banding patterns of the immunoaffinity-purified proteins looked similar using either a 4F6 or 4H3 column (**Figure 2A**). To identify which bands were specifically recognized, the immunoaffinity-purified materials were analyzed by western blot by staining independently with each mAb. **Figure 2B** shows that 4F6 recognizes a single band around 47 kDa under reducing conditions in separated proteins immunoaffinity-purified by either 4F6 (**Figure 2B**, lane 1) or 4H3 (**Figure 2B**, lane 2), and 4H3 recognized multiple bands around 100 kDa under non-reducing conditions. HMW bands recognized only under non-reducing conditions were likely non-specific reaction with secondary antibody because these bands were also visible in the negative controls (**Figure 2B**, PBS/T). By LC-MS/MS analyses we identified the 4F6 immunoprecipitates as PfRAP1 and PfRAP2 (**Figure 2C**). Taken together, the target antigens of both mAbs are the described LMW rhoptry protein complex (**Table 1**, **Supplementary Tables 1****,** **2**, and **Figure 2C**).

**Table 1:** LC-MS/MS analysis of immunoaffinity-purified proteins with each monoclonal antibody from *Plasmodium falciparum* schizont lysates.

mAbs	Protein	MW (kDa)	%^a^	PlasmoDB ID	Peptide sequences identified^b^	Score^c^
4F6	PfRAP2	46.7	43	PF3D7_0501600	^52^LSMWVYFIYNHFSSADELIK^71^//^314^QFDYALFHKTYSIPNLK^330^	996
	PfRAP1	90.0	41	PF3D7_1410400	^181^SASVAGIVGADEEAPPAPKNTLTPLEELYPTNVNLFNYKYSLNNMEENIN	1420
					ILKNEGDLVAQKEEFEYDENMEK^253^//^712^MKTDMLSLQNEESK^725^	
1C2	PfRhopH2	162.6	27	PF3D7_0929400	^52^YLYMDEYLSEGDKATFEK^69/^/^1193^LFVTEGTLEYLLLDK^1207^	1792
	Clag9	160.4	9	PF3D7_0935800	^35^SILDNDELYNSLSNLENLLLQTLEQDELK^63^//^1259^ENVVQEVQEDK^1269^	317
	PfRhopH3	104.8	21	PF3D7_0905400	^111^EYEEPFVNPVMK^122^//^824^TTDNTYKEMEELEEAEGTSNLK^845^	622
4E6	PfRhopH3	104.8	36	PF3D7_0905400	^52^GNGPDAGSFLDFVDEPEQFYWFVEHFLSVK^81^//^793^STSAASTSDEISGSEGPS	1063
					TESTSTGNQGEDKTTDNTYKEMEELEEAEGTSNLK^845^	
	Clag3.1	167.2	20	PF3D7_0302500	^78^LILESLEKDK^87^//^1390^MNEADSADSDDEKDSDTPDDELMISR^1415^	992
	Clag3.2	167.5	15	PF3D7_0302200	^37^NENANVNTPENLNK LLNEYDNIEQLK^62^//	858
					^964^TMFAAFQMLFSTMLSNNVDNLDK^986^	
	Clag9	160.4	12	PF3D7_0935800	^35^SILDNDELYNSLSNLENLLLQTLEQDELKIPIMK^68^//^1244^EGAYEEAMVSR^1254^	483
1G5	PfRAMA	103.6	11	PF3D7_0707300	^589^YLDDLIDEEQTIKDAVK^605^//^735^INDELLTDQGPNEDTLLENNNK^756^	401
	PfRON3	263.0	3	PF3D7_1252100	^374^NLGTGFFDFSNSLFK^388^//^1558^FLADSNIPSIPYQGFSVR^1575^	156

^a^Percent peptide coverage (%) is shown for each protein. All regions covered by identified peptides are shown in red text in **Supplementary Table 1**. ^b^Representative two peptide sequences with higher scores among all the identified peptides are shown. The number represents the position at the N- and C-terminus of each peptide (“//”). ^c^Score is a sum of the scores of all the identified peptides. Each peptide score is −10 × Log_10_(P), where P is the probability that the observed match is a random event. Peptide scores greater than 27 indicate identity or extensive homology (P < 0.05). RAP, rhoptry-associated protein; RhopH, high-molecular mass rhoptry protein complex; Clag, cytoadherence-linked asexual gene; RAMA, rhoptry-associated membrane antigen; RON, rhoptry neck protein.

### Monoclonal Antibodies 1C2 and 4E6 Recognize the High-Molecular Weight PfRhopH Complex Proteins

MAb 1C2 recognized a distinct antigen from that identified by 4E6 in western blots of parasite lysates (**Supplementary Figure 1**). A single major band around 140 kDa was visible in SDS-PAGE in the immunoaffinity-purified proteins using a 1C2 column, whereas triple major bands were visible in the immunoaffinity-purified proteins using a 4E6 column (**Figure 2D**). The immunoaffinity-purified materials were analyzed by western blot by independently staining with each mAb. **Figure 2E** shows that 1C2 recognized a single band around 140 kDa under reducing conditions of separated proteins immunoaffinity-purified by either 1C2 **(****Figure 2E**, 1C2, lane 1) or 4E6 (**Figure 2E**, 1C2, lane 2). In contrast, we could not identify target antigen bands by western blot with 4E6 staining, perhaps because of the lower reactivity of the mAb 4E6 under reducing conditions (**Figure 2E**, 4E6). By LC-MS/MS analyses, we identified that mAb 1C2 dominantly recognized PfRhopH2 (**Figure 2F**, lane 1C2) and associated PfRhopH complex partners Clag 9 and PfRhopH3 as minor bands. In contrast, mAb 4E6 dominantly recognized PfRhopH3 (**Figure 2F**, lane 4E6) and associated PfRhopH complex partners as minor bands (Clag 3.1, Clag 3.2, and Clag 9). Taken together, the target antigens of both mAbs are within the HMW rhoptry protein complex (**Table 1**, **Supplementary Tables 1****,** **2**, and **Figure 2F**).

### Monoclonal Antibody 1G5 Recognizes the PfRAMA Protein

MAb 1G5 recognized a major band around 60 kDa with multiple bands between 170 and 30 kDa in western blots of parasite lysates (**Supplementary Figure 1**). A single major band around 60 kDa was also visible in SDS-PAGE of the immunoaffinity-purified proteins using a 1G5 column (**Figure 2G**). The immunoaffinity-purified materials were analyzed by western blot and 1G5 staining. **Figure 2H** shows that 1G5 recognizes a major band around 60 kDa and at least seven additional bands between 100 kDa and 30 kDa, suggesting that those bands are proteolytically cleaved fragments from a single molecule. The SDS-PAGE results using a 10% gel at APRO SCIENCE showed that a major band around 60 kDa and a high-molecular weight band were identified (**Figure 2I**). LC-MS/MS determined that 1G5 recognizes PfRAMA (**Figure 2I**) and that an associated PfRON3 protein is also identified. Taken together, PfRAMA was the target antigen of mAb 1G5, and these data suggest that PfRAMA forms a protein complex with PfRON3 (**Table 1**, **Supplementary Tables 1****,** **2**, and **Figure 2I**).

### Complex Formation Between PfRAMA and PfRON3 Proteins in the Early Schizont Stage

**Figure 3:**
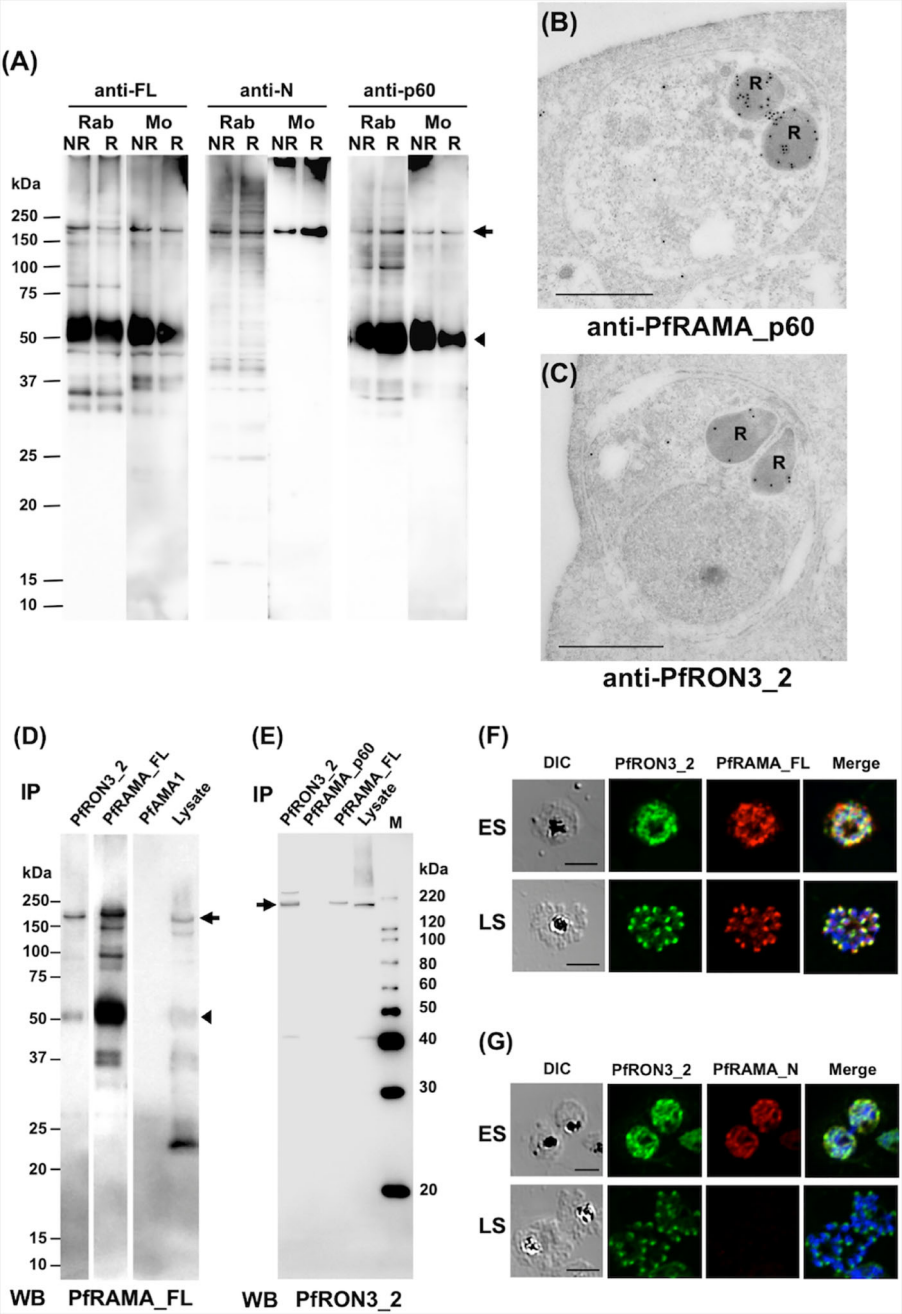
**(A)** Specificity of anti-PfRAMA antibodies by western blot analyses. Proteins from schizont-rich parasites were extracted and separated by 12.5% SDS-PAGE under non-reducing (NR) or reducing (R) conditions. Using either anti-PfRAMA_FL, anti-PfRAMA_N, or anti-PfRAMA_p60 antibodies obtained from rabbits (Rab) and mice (Mo), a band of approximately 170 kDa (arrow) was detected as a signal of PfRAMA_FL and a 60-kDa band (arrowhead) was detected as a signal of PfRAMA_p60. **(B)** PfRAMA localization by IEM is shown. A representative image out of eight independent sections is shown of a merozoite in a schizont-infected erythrocyte probed with rabbit anti-PfRAMA_p60 antibody and subsequently with a secondary antibody conjugated with gold particles. The black dots indicate signals from gold particles localized in the rhoptry bulb. R, rhoptry. **(C)** PfRON3 localization shown by IEM. A representative image out of 16 independent sections is shown of a merozoite in a schizont-infected erythrocyte probed with rabbit anti-PfRON3_2 antibody and subsequently with a secondary antibody conjugated with gold particles. The black dots indicate signals from gold particles localized in the rhoptry bulb. R, rhoptry. Bars = 500 nm. **(D)** PfRAMA_FL interacts with PfRON3. NP-40 extracts of schizont-rich parasites (Lysate) were immunoprecipitated (IP) with rabbit sera against PfRON3 (anti-PfRON3_2), PfRAMA (anti-PfRAMA_FL), or PfAMA1 (anti-PfAMA1), then stained with mouse antisera (WB) against PfRAMA_FL. This panel is a representative result of two independent experiments. **(E)** PfRAMA_FL but not PfRAMA_p60 interacts with PfRON3. NP-40 extracts of schizont-rich parasites (Lysate) were immunoprecipitated (IP) with rabbit sera against PfRON3 (anti-PfRON3_2), PfRAMA (anti-PfRAMA_p60), or PfRAMA (anti-PfRAMA_FL), then stained with mouse antisera (WB) against PfRON3_2. M, molecular weight marker. This panel is a representative result of two independent experiments. **(F)** Co-localization of PfRON3 and PfRAMA. Immature early schizont (ES) or mature late schizont (LS) stage parasites were dual-labeled with rabbit antibodies against PfRON3_2 and mouse antibodies against PfRAMA_FL. Nuclei were visualized with DAPI in merged images shown in the right most panels. DIC, differential interference contrast microscopy. Merge, the image created by merging the IFA and nuclear-staining images. Bars represent 5 μm. **(G)** Co-localization of PfRON3 and PfRAMA. Immature early schizont (ES) or mature late schizont (LS) stage parasites were dual-labeled with rabbit antibodies against PfRON3_2 and mouse antibodies against PfRAMA_N. Nuclei were visualized with DAPI in merged images shown in the right most panels. DIC, differential interference contrast microscopy. Merge, the image created by merging the IFA and nuclear-staining images. Bars represent 5 μm.

To confirm the specificity of polyclonal anti-RAMA antibodies western blot analyses of schizont-rich parasite lysates were performed under non-reducing (NR) and reducing (R) conditions. Rabbit and mouse anti-PfRAMA_FL and anti-PfRAMA_p60 antibodies recognized both PfRAMA_FL at the expected molecular weight of 170 kDa (**Figure 3A**, arrow) and PfRAMA_p60 at the expected molecular weight of 60 kDa (**Figure 3A**, arrowhead); however, anti-PfRAMA_N antibodies recognized only PfRAMA_FL (**Figure 3A**, arrow). In addition, anti-PfRAMA_FL rabbit antibodies recognized the rhoptry bulb by IEM (**Figure 3B**), and confirmed that the anti-PfRAMA antibodies specifically recognized PfRAMA. We also confirmed the specificity of the anti-PfRON3_2 rabbit antibody as rhoptry bulb localization (**Figure 3C**).

Immunoprecipitation assays were performed to validate the PfRAMA interaction with PfRON3. First, we immunoprecipitated PfRON3, PfRAMA, and PfAMA1 proteins in schizont-rich parasite lysates using rabbit anti-PfRON3_2, anti-PfRAMA_FL, and anti-PfAMA1 antibodies. By western blot analyses the immunoprecipitates were probed with mouse anti-PfRAMA_FL antibodies (**Figure 3D**). We observed that anti-PfRON3_2 antibody could coimmunoprecipitate both PfRAMA_FL (**Figure 3D**, arrow) and PfRAMA_p60 (**Figure 3D**, arrowhead). The signal intensity of the PfRAMA_FL band was relatively stronger than that of PfRAMA_p60 in PfRON3_2 immunoprecipitates, suggesting that PfRON3 formed a more stable complex with PfRAMA_FL than PfRAMA_p60. By comparison, anti-PfAMA1 antibodies as a negative control did not immunoprecipitate PfRAMA. As a reverse experiment we immunoprecipitated PfRON3 and PfRAMA proteins in the same parasite lysates using rabbit anti-PfRON3_2, anti-PfRAMA_p60, and anti-PfRAMA_FL antibodies and probed with mouse anti-PfRON3_2 antibodies as above (**Figure 3E**). We observed that anti-PfRAMA_FL antibody could coimmunoprecipitate PfRON3 (**Figure 3E**, arrow); however, anti-PfRAMA_p60 could not (**Figure 3E**). These results confirmed that PfRAMA (except for the PfRAMA_p60 region which is known to associate with PfRAP1, PfRhopH3, and PfSortilin) formed a protein complex with PfRON3.

IFA was performed to investigate in which developmental stages PfRAMA interacts with PfRON3. By immunostaining with anti-PfRON3_2 and anti-PfRAMA_FL antibodies, PfRON3, and PfRAMA were colocalized mostly in the cytoplasm in early schizonts (**Figure 3F**, ES), and in a patchy pattern in each merozoite in late schizonts (**Figure 3F**, LS) suggesting rhoptry localization. In contrast, when PfRAMA was immunostained with anti-PfRAMA_N antibodies, PfRON3, and PfRAMA also colocalized mostly in the cytoplasm in early schizonts (**Figure 3G**, ES); however, a lack of staining in late schizonts (**Figure 3G**, LS) suggested that the PfRAMA_N region was not present in the merozoite rhoptry in mature schizonts. These results suggest that the PfRAMA_N region may form a protein complex with PfRON3 in the early schizont stage.

## Discussion

Identification of novel apical organellar proteins of merozoite are essential for understanding merozoite invasion into erythrocytes as well as providing new vaccine candidates for study. Here we generated 12 mAbs which recognize merozoite apical organelles. Immunoaffinity-purification combined with LC-MS/MS identified target antigens of 5 mAbs as PfRAP1, PfRAP2, PfRhopH2, PfRhopH3, and PfRAMA. Although these five antigens are known rhoptry bulb proteins (Counihan et al., 2013), the identification of a novel PfRAMA/PfRON3 rhoptry protein complex in the *P. falciparum* merozoite is emphasized.

PfRAMA is a rhoptry bulb protein which is expressed relatively early before the *de novo* formation of rhoptries. After proteolytic cleavage a PfRAMA_p60 fragment is formed and localizes in the merozoite rhoptry bulb in the late schizont stage (Smythe et al., 1988; Topolska et al., 2004). Thereafter, PfRAMA_p60 localizes to the rhoptry but not on the surface of the free merozoite. When the merozoite attaches to the erythrocyte, the discharged PfRAMA_p60 binds to the erythrocyte surface. Subsequently, PfRAMA_p60 is localized in the PV membrane during merozoite invasion (Smythe et al., 1988; Topolska et al., 2004).

To elucidate the function of PfRAMA two studies demonstrated by fluorescent resonance energy transfer (FRET) and immunoprecipitation that PfRAMA interacts with both PfRAP1 and PfRhopH3 (Topolska et al., 2004; Richard et al., 2009). Recently the PfRAMA-PfRAP1 complex was also suggested as a cargo for the *Plasmodium* orthologue of sortilin (Hallee et al., 2018b). To further investigate the role of PfRAMA, Sherling et al., (2019) generated a PfRAMA conditional knockdown parasite line. Contrary to previous findings (Topolska et al., 2004; Richard et al., 2009; Hallee et al., 2018b), the PfRAMA knockdown parasites presented correct trafficking of PfRAP1 and PfRhopH3. In addition, several other rhoptry bulb proteins, such as PfRAP2, PfRh5, Clag3.1, and PfRhopH2 also localized correctly to the rhoptry in the transgenic parasites. Therefore, their findings were inconsistent with the proposed rhoptry bulb-specific protein escorter role of PfRAMA (Hallee et al., 2018b). Furthermore, although the knockdown parasites showed that some RON proteins—PfRON2, PfRON3, and PfRON4—were diminished in mature schizonts, the rhoptry neck proteins PfRON12 and Rh2b were normally localized in the rhoptry (Sherling et al., 2019). While PfRON3 is now known as a rhoptry body protein (Ito et al., 2011) (**Figure 3C**), they suggested that the mislocalization of the above RON proteins may be due to abnormal rhoptry neck biogenesis. In this study we identified by immunoprecipitation an interaction of PfRON3 with PfRAMA_FL but not with PfRAMA_p60 (**Figures 3D, E****)**. We also showed their colocalization when stained with anti-PfRAMA_FL and PfRON3 antibodies but not with anti-PfRAMA_N antibodies in the mature schizont stage (**Figures 3F, G****)**. These results suggest that the association between PfRAMA and PfRON3 occurs in the immature schizont stage, and thereafter the two proteins dissociate when the N-terminal region of PfRAMA (downstream from the PfRAMA_N region) is proteolytically degraded in the mature schizont (Smythe et al., 1988; Topolska et al., 2004). In addition, we previously reported that PfRON3 interacts with PfRON2 and PfRON4, but not with PfAMA1 (Ito et al., 2011), suggesting that a portion of PfRON3 is involved in the formation of a RON complex (PfRON2, 3, and 4), but not in the moving junction complex (PfRON2, 4, 5, and PfAMA1) (Ito et al., 2011). Taken together, the absence of PfRAMA affects the trafficking of its associated RONs, and this could potentially explain the abnormal rhoptry neck biogenesis as observed by Sherling et al., (2019).

To predict the PfRON3 associating region in PfRAMA, the PfRAMA knockdown parasite generated by Sherling et al., (2019) provided us with useful information. The knockdown parasite with abnormal PfRON3 trafficking resulted in expression of the N-terminal 220 residues of the protein but lacking the C-terminal region spanning aa V_315_–S_840_. This C-terminal 526-residue protein was previously shown to interact with both PfRAP1 and PfSortilin (Topolska et al., 2004; Richard et al., 2009; Hallee et al., 2018a; Hallee et al., 2018b). Additional evidence is that our anti-PfRAMA_p60 (K_482_ to F_758_) antibodies failed to immunoprecipitate PfRON3 (**Figure 3E**). Taken together, the PfRAMA residues spanning aa Q_221_-E_481_ may be important for the trafficking of PfRON3 to the rhoptries. Further investigation will be required as to whether PfRAMA and PfRON3 interact directly or indirectly, such as by using a surface plasmon resonance approach with recombinant proteins.

## Data Availability Statement

The raw data supporting the conclusions of this article will be made available by the authors, without undue reservation.

## Ethics Statement

Ethical review and approval was not required for the animal study because all the immunization for mouse monoclonal antibodies and rabbit polyclonal antibodies were done at Kitayama Labes company (Ina, Japan) under their ethics standards, and we purchased the generated antibodies from the Kitayama Labes company. So our IRB approval was not needed.

## Author Contributions

TT and E-TH conceived and designed the experiments. DI, J-HC, ET, TH, AT, and E-TH conducted experiments. DI, J-HC, ET, HO, ST, AT, E-TH, and TT analyzed the data. DI, J-HC, ET, E-TH, and TT wrote the manuscript. All authors contributed to the article and approved the submitted version.

## Funding

This work was supported in part by JSPS KAKENHI (Grant Nos. JP17H06873, JP18H02651, JP18K19455, JP19K22535, and JP20H03481) and Takeda Science Foundation. This work was also supported in part by the National Research Foundation of Korea Grant funded by the Korean Government (2015R1A4A1038666 and 2017R1A2A2A05069562). The funders had no role in the study design, data collection and analysis, decision to publish, or preparation of the manuscript.

## Conflict of Interest

The authors declare that the research was conducted in the absence of any commercial or financial relationships that could be construed as a potential conflict of interest.

The reviewer DW declared a past co-authorship with several of the authors ET and TT to the handling editor.

## Supplementary Material

Supplementary Figure 1 Characterization and isotyping of each monoclonal antibody reacting with the apical end of *P. falciparum* merozoites. To characterize target antigens recognized by the 12 mAbs, the mAb isotypes were determined using the culture supernatant and each mAb was purified from mouse ascitic fluid using a MAbTrap kit (GE Healthcare). The summary of the western blot analyses (12.5% SDS-PAGE gel) and isotyping are presented in the right side of this figure. MAb name with isotype in parenthesis and reacted bands are shown in kDa. N/A, unable to obtain purified mAbs; Negative, no band detected in western blot analysis; PBS/T, negative control stained with PBS/T; NR, nonreducing condition; and R, reducing condition.

## Abbreviations

BSA, bovine serum albumin; DAPI, 4′,6-diamidino-2-phenylindole; EDTA, ethylenediaminetetraacetic acid; ELISA, enzyme-linked immunosorbent assay; IFA, immunofluorescence assay; IgG, immunoglobulin G; MAb, monoclonal antibody; PBS, phosphate buffered saline; SDS-PAGE, sodium dodecyl sulfate- polyacrylamide gel electrophoresis; GPI, glycosylphosphatidylinositol; Pf, *P. falciparum*; PV, parasitophorous vacuole; AMA1, apical merozoite protein 1; RAMA, rhoptry-associated membrane antigen; RAP, rhoptry-associated protein; RON, rhoptry neck protein; RhopH, high-molecular weight rhoptry protein; Clag, cytoadherence-linked asexual gene; WGCFS, wheat germ cell-free protein synthesis system.
