# Supplementary figures and images for: Identification of a Novel RAMA/RON3 Rhoptry Protein Complex in Plasmodium falciparum Merozoites
Source: Front Cell Infect Microbiol. 2021 Jan 18;10:605367. doi: 10.3389/fcimb.2020.605367 (PMC7848174; doi:10.3389/fcimb.2020.605367)

### *P. falciparum* monoclonal antibodies

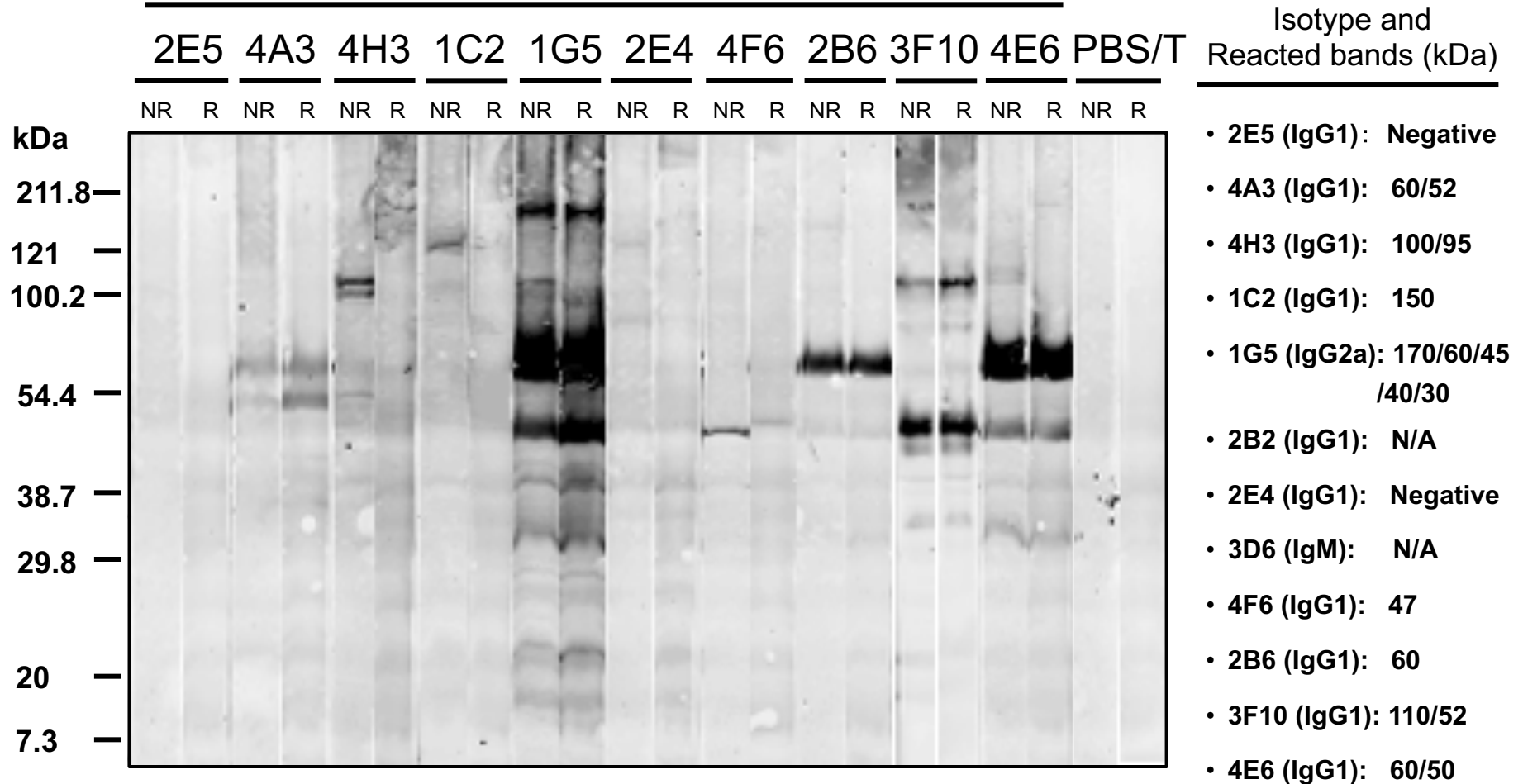

Supplement: Supplementary Figure 1 — Characterization and isotyping of each monoclonal antibody reacting with the apical end of P. falciparum merozoites. To characterize target antigens recognized by the 12 mAbs, the mAb isotypes were determined using the culture supernatant and each mAb was purified from mouse ascitic fluid using a MAbTrap kit (GE Healthcare). The summary of the western blot analyses (12.5% SDS-PAGE gel) and isotyping are presented in the right side of this figure. MAb name with isotype in parenthesis and reacted bands are shown in kDa. N/A, unable to obtain purified mAbs; Negative, no band detected in western blot analysis; PBS/T, negative control stained with PBS/T; NR, nonreducing condition; and R, reducing condition. [file DataSheet_1.pdf]
