# Supplementary material for: Identification of a Novel RAMA/RON3 Rhoptry Protein Complex in Plasmodium falciparum Merozoites
Source: Front Cell Infect Microbiol. 2021 Jan 18;10:605367. doi: 10.3389/fcimb.2020.605367 (PMC7848174; doi:10.3389/fcimb.2020.605367)
Supplement: Supplementary file 2 [file Table_1.docx]

**SUPPLEMENTARY TABLE 1.** **Location of peptides identified by LC-MS/MS analysis of SDS-PAGE gels.**

All regions covered by identified peptides are shown in red. Representative two peptide sequences with higher scores among the identified peptides listed in Table 1 are underlined.

**1. 4F6 mAb:**

**- Rhoptry-associated protein 2 [*Plasmodium falciparum* 3D7], RAP2, PF3D7_0501600**

1 MGLKFYVLVF LILCLKNVVK GDKCETEFSK LYPESNSLTG LIYAHTAHVH

51 KLSMWVYFIY NHFSSADELI KYLEKTNINT LENSDHTCFA RAVTLYLFYY

101 YLKDIKSMLS TDDYQSFFKN KFKDINPLFI NDFILILNDK KFMENLDLYI

151 MKESEREHLV IKKNPFLRVL NKASTTTHAT YKSNPYFIVG SRVHTPYKDY

201 LGDFNKYTEI SVLNYVRDYN FLIYAGSREN YYNSDIAGPA RSVNNVISKN

251 KTLGLRKRSS SLALVGTNNN DPIFAYCEKD NKSEYYGTPD DLITSFFSII

301 KTKMLNSHKT FLRQFDYALF HKTYSIPNLK GFRFLKHLFQ KKNLVNFVGM

351 YENHVSTEIN FLAEDFVELF DVTMDCYSRQ YSNRAAENFK AIRELNVL

**- Rhoptry-associated protein 1 [*Plasmodium falciparum* 3D7], RAP1, PF3D7_1410400**

1 MSFYLGSLVI IFHVLFRNVA DGINVNGDNN YGKTIINNDF NFDDYNYWTP

51 INKKEFLNSY EDKFSSESFL ENKSSVDDGN INLTDTSTSN KSSKKGHGRS

101 RVRSASAAAI LEEDDSKDDM EFKASPSVVK TSTPSGTQTS GLKSSSPSST

151 KSSSPSNVKS ASPHGESNSS EESTTKSSKR SASVAGIVGA DEEAPPAPKN

201 TLTPLEELYP TNVNLFNYKY SLNNMEENIN ILKNEGDLVA QKEEFEYDEN

251 MEKAKQDKKK ALEKIGKESD EEPFMFSENK FLENQVKERN VAGSFSRFFS

301 KLNPFKKDEV IEKTEVSKKT FSGIGFNLTE KEAKVLGVGV TYQEYPETML

351 YNCPNNSNLF DTIESLQGRI IDIKKRESMI STTFEQQKEC LKNMGVLDLE

401 LNDTQCKFGT CIGSFGEHHL RLYEFENDLL KFHPNIDYLT LADGYKLQKN

451 DIYELSHVNF CLLNPKTLEE FLKKKEIKDL MGGDDLIKYK ENFDNFMSIS

501 ITCHIESLIY DDIEASQDIA AVLKIAKSKL HVITSGLSYK ARKLVYKIYS

551 EIQKNPDELY EKLTWIYDNI YMIKRYYTAY ALEGVCSYLE HDKSQMYTEL

601 HIYNKIVDSV RYYSSCFKNV IVYNAIISGI HEKIKHFLKL VPRHNFLLDY

651 HFNSIFEKEI KPAKKYSTSH IYFDPTVASY AYYNLDRRTM VTIINDYFEA

701 KKKELTVIVS RMKTDMLSLQ NEESKIPNDK SANSKLATRL MKKFKAEIRD

751 FFKEMRIQYA KLINIRYRSH LKKNYFAFKR LD

**2. 1C2 mAb:**

**- High molecular weight rhoptry protein-2 [*Plasmodium falciparum* 3D7], RhopH2, PF3D7_0929400**

1 MIKVTIFLLL SIFSFNLYGL ELNEKVSIKY GAEQGVGSAD SNTKLCSDIL

51 KYLYMDEYLS EGDKATFEKK CHNVIGNIRN TFSNKNTIKE GNEFLMSILH

101 MKSLYGNNNN NNAGSESDVT LKSLYLSLKG SQNTEGESEV PSDDEINKTI

151 MNFVKFNKYL LDNSNDIKKV HDFLVLTSQS NENLLPNKEK LFEQIVDQIK

201 YFDEYFFASG GKIKVKKGYL KYNFLDIYKQ PVCSAYLHLC SRYYESVSIY

251 IRLKKVFNGI PAFLDKNCRK VKGEEFKKLM DMELKHNHIV ERFDKYIISD

301 DLYYVNMKVF DLKNVDKIQV SKIDDINNLN IYEHKETMHL SAKNLSRYID

351 IKKELNDEKA YKQLMSAIRK YVTTLTKADS DITYFVKQLD DEEIERFLID

401 LNFFLYNGFL RITEDKHLIN ADDVSPSYIN LYRSNNIVAL YILKTQYEEN

451 KLSEYRAHKF YRRKRVSNIT NDMIKKDFTQ TNALTNLPNL DNKKTTEYYL

501 KEYENFVENF QPDLHDIMKL QLFFTMAFKD CNVNQNFTET SKKLWFDLLY

551 AYDKFGWFYI HPNEVINSIN KTDFVRHVLV SRNFLLKNND QLTFLETQVA

601 KIVEIINLSL EVDKSPDSLD FSIPMNFFNH KNGYHVMNDD KLKLLTSYEY

651 IDSIANNYFF LSEYKNDVFR TGNNFKLYFN LPNIYSLAYQ LFNELAININ

701 VITNVPLKKY LKYNASYAYF TLMNMIGKNH DIYSKGSRFV YASYILGLVF

751 FIESHIDIAR LKPKDFFFMK QSLPIIDHVY HKDLKTLKKN CTLLTDFMKI

801 NKNSQNYSLT HTEEMIKILG LLTVTLWAKE GKKSVYYDDD VSLYRKLMVS

851 CVFNGGETIQ EKLANNIEKS CDISQYGIKS KNLKDMIDIN LSIHKWNPAE

901 IEKLAYSFVL SCKMQKLMYK PMNVEKLPLE DYYKLSLAPD MVKTYHCYKL

951 GKQAAELLES IILKKKFVRF RVTDAIDVYD FFYIKKVLSS RIKKEYNEFL

1001 QDKRAFEKKE LETILNNSPF SEEQTMKLIN SYECHWFTSY ENFRILWMHA

1051 SSNLGTGTYL KNFFSELWQN IRFLFKSKLK IRDMEYFSGD ISQMNLLDYY

1101 SPMVHSESHC QEKMQVLFIT LRDSKEENRS EIAQKVKSAY YQCKLDYYKN

1151 HHSDFIHRIH PNDFLNNKVY VLKQPYYLMS NVPLNNPKKV SRLFVTEGTL

1201 EYLLLDKINI PECFGPCTKL HFNKVVIKES KQRIYDMTIN NALVPEIQPY

1251 NRRKYMTIYI NEAYIKNIVS DALTSEEIKR HDIQKGNIKI CMGKSTYLTE

1301 PILTEEHFNL THKPVYDFSS VKHNLKVFHM KNEHLVSEDP NDDCFINYPL

1351 ATINLDISDP YKEISEDLIK NLYILKSS

**- Cytoadherence linked asexual protein; CLAG9 [*Plasmodium falciparum* 3D7], PF3D7_0935800**

1 MIIWFIQPTI FYIIFILARN IQCTYKGDNI NEIKSILDND ELYNSLSNLE

51 NLLLQTLEQD ELKIPIMKGD LDKYLNMSNF KILNELNADG AEKPYIIPTS

101 NCSANDIVKY EHTLKTQITL EYKPEISDML KRKNIVVRTL KIIKFMQTPM

151 SAYKNTNNIK QSLLEMNKLF TNKEKKLNEH TINALRLRDR IFNTNNLTHR

201 SIKKGISTYM PIDTKSDIID YDDLLFTNHP SIELMENLDK LANYYHIGIF

251 NMIGSHYIAV GHFITLKLAL KNYKKYFEIG SLKYLNWQSI LKFNQSDRFK

301 VLDLICDESS WYEGQMKRRE QYLKNNIFST SEECSVLEFL IHHMNKYQME

351 LYSKMHKLSL NVQIYLENKH LKEKFLQFMC RSRKECNIYE SDRFKQEQEK

401 GIEFHDNNNY KFSQENDPVS KVVDPFNLFT NYFYFIKYYS VFNSDHIIYM

451 HLLNFVGVLN GNNNAYVSSL YLPGYYNAIQ LSYKDQVGLK ELYQNLVKCV

501 EKCYIRNRKN RSFSHRIASI FRHKKFDSSK CSICEGTLLY INDQSQDKMS

551 MAQKFYIFVT KILKVNNISS FITNMNIYED YSNYLMHDLN WYTFLFLFRM

601 TTYKDIPNYS ISNAMYLNIK DEDDTKRTMV TFQWMPSTIK RMHNYRIRKY

651 ISIYLLEELE KLIDNKLIEK LKKCITFLIH LNAFLQLDFF SYLNETPANL

701 QHPFPISMMI EARFKDWFIQ YLTGFFFINY DDANTRYNMP ENMKRGTFIP

751 PKYSKWNIHL KRFIDEAFLM YFNQKHALTL FKYHNPYNIS NKIMLMRDTF

801 ELYTKNYDQL IFGADIMLLR KTFSCTPMST KVWDRVKYYL HNIIGNPINF

851 YKHGLIYAYT LNKAMLKEVV NDFFVIYKMN KDLFSETSFL QTVYLLFKKI

901 QGTYFSHRRN DDVSMNNIFM FNVEKNYSKM SQADREKEIH ESMASRFFAK

951 NLFTVFQMMF VIQISNDVDK LDRIYGKADM LRLSVHDEPF LRFAYAYYGS

1001 MYDKLTNVFF PMNIKKPTIQ LKYGKTFIMA NLYYLCSVLF SMYNLNNLGL

1051 LCEYQAIGSA NFHSYKKMSQ FIDKKFIPLV FYTLKARTEG IIGKEWYKMV

1101 FNDFDGKSMA NTWPYFGYYM GGNMLYRNIL YFPNHLPEEL RKQTKGVELQ

1151 QPEYEPSVHS IDWQVGYAIS HGLSLSFFTF GMMKAYAYFE NVIFFLRNSI

1201 RIFDRFYSIL ENYVCMYIKR LFNKLTVDKL LKAMSRAYTS TKKEGAYEEA

1251 MVSRVRNKEN VVQEVQEDKG TDITPLPTFD IMDSKQNTNY MYNDNEDYFD

1301 DLDDNEQFLN SKDLLYYDDG IDRTKRYELI PLQRYRYDPF

- **High molecular weight rhoptry protein-3 [*Plasmodium falciparum* 3D7], RhopH3, PF3D7_0905400**

1 MRSKHLVTLF IITFLSFSTV KVWGKDVFAG FVTKKLKTLL DCNFALYYNF

51 KGNGPDAGSF LDFVDEPEQF YWFVEHFLSV KFRVPKHLKD KNIHNFTPCL

101 NRSWVSEFLK EYEEPFVNPV MKFLDKEQRL FFTYNFGDVE PQGKYTYFPV

151 KEFHKYCILP PLIKTNIKDG ESGEFLKYQL NKEEYKVFLS SVGSQMTAIK

201 NLYSTVEDEQ RKQLLKVIIE NESTNDISVQ CPTYNIKLHY TKECANSNNI

251 LKCIDEFLRK TCEKKTESKH PSADLCEHLQ FLFESLKNPY LDNFKKFMTN

301 SDFTLIKPQS VWNVPIFDIY KPKNYLDSVQ NLDTECFKKL NSKNLIFLSF

351 HDDIPNNPYY NVELQEIVKL STYTYSIFDK LYNFFFVFKK SGAPISPVSV

401 KELSHNITDF SFKEDNSEIQ CQNVRKSLDL EVDVETMKGI AAEKLCKIIE

451 KFILTKDDAS KPEKSDIHRG FRILCILIST HVEAYNIVRQ LLNMESMISL

501 TRYTSLYIHK FFKSVTLLKG NFLYKNNKAI RYSRACSKAS LHVPSVLYRR

551 NIYIPETFLS LYLGLSNLVS SNPSSPFFEY AIIEFLVTYY NKGSEKFVLY

601 FISIISVLYI NEYYYEQLSC FYPKEFELIK SRMIHPNIVD RILKGIDNLM

651 KSTRYDKMRT MYLDFESSDI FSREKVFTAL YNFDSFIKTN EQLKKKNLEE

701 ISEIPVQLET SNDGIGYRKQ DVLYETDKPQ TMDEASYEET VDEDAHHVNE

751 KQHSAHFLDA IAEKDILEEK TKDQDLEIEL YKYMGPLKEQ SKSTSAASTS

801 DEISGSEGPS TESTSTGNQG EDKTTDNTYK EMEELEEAEG TSNLKKGLEF

851 YKSSLKLDQL DKEKPKKKKS KRKKKRDSSS DRILLEESKT FTSENEL

**3. 4E6 mAb:**

**- High molecular weight rhoptry protein-3 [*Plasmodium falciparum* 3D7], RhopH3, PF3D7_0905400**

1 MRSKHLVTLF IITFLSFSTV KVWGKDVFAG FVTKKLKTLL DCNFALYYNF

51 KGNGPDAGSF LDFVDEPEQF YWFVEHFLSV KFRVPKHLKD KNIHNFTPCL

101 NRSWVSEFLK EYEEPFVNPV MKFLDKEQRL FFTYNFGDVE PQGKYTYFPV

151 KEFHKYCILP PLIKTNIKDG ESGEFLKYQL NKEEYKVFLS SVGSQMTAIK

201 NLYSTVEDEQ RKQLLKVIIE NESTNDISVQ CPTYNIKLHY TKECANSNNI

251 LKCIDEFLRK TCEKKTESKH PSADLCEHLQ FLFESLKNPY LDNFKKFMTN

301 SDFTLIKPQS VWNVPIFDIY KPKNYLDSVQ NLDTECFKKL NSKNLIFLSF

351 HDDIPNNPYY NVELQEIVKL STYTYSIFDK LYNFFFVFKK SGAPISPVSV

401 KELSHNITDF SFKEDNSEIQ CQNVRKSLDL EVDVETMKGI AAEKLCKIIE

451 KFILTKDDAS KPEKSDIHRG FRILCILIST HVEAYNIVRQ LLNMESMISL

501 TRYTSLYIHK FFKSVTLLKG NFLYKNNKAI RYSRACSKAS LHVPSVLYRR

551 NIYIPETFLS LYLGLSNLVS SNPSSPFFEY AIIEFLVTYY NKGSEKFVLY

601 FISIISVLYI NEYYYEQLSC FYPKEFELIK SRMIHPNIVD RILKGIDNLM

651 KSTRYDKMRT MYLDFESSDI FSREKVFTAL YNFDSFIKTN EQLKKKNLEE

701 ISEIPVQLET SNDGIGYRKQ DVLYETDKPQ TMDEASYEET VDEDAHHVNE

751 KQHSAHFLDA IAEKDILEEK TKDQDLEIEL YKYMGPLKEQ SKSTSAASTS

801 DEISGSEGPS TESTSTGNQG EDKTTDNTYK EMEELEEAEG TSNLKKGLEF

851 YKSSLKLDQL DKEKPKKKKS KRKKKRDSSS DRILLEESKT FTSENEL

**- Cytoadherence linked asexual protein [*Plasmodium falciparum* 3D7] , CLAG3.1, PF3D7_0302500**

1 MVSFFKTPIF ILIIFLYLNE KVICSINENQ NENDTISQNV NQHENINQNV

51 NDNDNIEQLK SMIGNDELHK NLTILEKLIL ESLEKDKLKY PLLKQGTEQL

101 IDISKFNKKN ITDADDETYI IPTVQSTFHD IVKYEHLIKE QSIEIYNSDI

151 SDKIKKKIFI VRTLKTIKLM LIPLNSYKQN NDLKSALEEL NNVFTNKEAQ

201 EESSPIGDHG TFFRKLLTHV RTIKENEDIE NKGETLILGD NKIDVMNSND

251 FFFTTNSNVK FMENLDDITN QYGLGLINHL GPHLIALGHF TVLKLALKNY

301 KNYFEAKSIK FFSWQKILEF SMSDRFKVLD MMCDHESVYY SEKKRRKTYL

351 KVDRSNTSME CNILEYLLHY FNKYQLEIIK TTQDTDFDLH GMMEHKYIKD

401 YFFSFMCNDP KECIIYHTNQ FKKEANEENT FPEQEEPNRQ ISAFNLYLNY

451 YYFMKRYSSY GVKKTLYVHL LNLTGLLNYD TRAYVTSLYL PGYYNAVEMS

501 FTEEKEFSKL FESLIQCIEK CHSDQARQIS KDSNLLNNIT KCDLCKGAFL

551 YANMKFDEVP SMLQKFYVYL TKGLKIQKVS SLIKTLDIYQ DYSNYLSHDI

601 NWYTFLFLFR LTSFKEIAKK NVAEAMYLNI KDEDTFNKTV VTNYWYPSPI

651 KKYYTLYVRK HIPNNLVDEL EKLMKSGTLE KMKKSLTFLV HVNSFLQLDF

701 FHQLNEPPLG LPRSYPLSLV LEHKFKEWMN SSPAGFYFSN YQNPYIRKDL

751 HDKVLSQKFE PPKMNQWNKV LKSLIECAYD MYFEQRHVKN LYKYHNIYNI

801 NNKLMLMRDS IDLYKNNFDD VLFFADIFNM RKYMTATPVY KKVKDRVYHT

851 LHSITGNSVN FYKYGIIYGF KVNKEILKEV VDELYSIYNF NTDIFTDTSF

901 LQTVYLLFRR IEETYRTQRR DDKISVNNVF FMNVANNYSK LNKEEREIEI

951 HNSMASRYYA KTMFAAFQML FSTMLSNNVD NLDKAYGLSE NIQVATSTSA

1001 FLTFAYVYNG SIMDSVTNSL LPPYAKKPIT QLKYGKTFVF SNYFMLASKM

1051 YDMLNYKNLS LLCEYQAVAS ANFYSAKKVG QFLGRKFLPI TTYFLVMRIS

1101 WTHAFTTGQH LISAFGSPSS TANGKSNASG YKSPESFFFT HGLAAEASKY

1151 LFFYFFTNLY LDAYKSFPGG FGPAIKEQTQ HVQEQTYERK PSVHSFNRNF

1201 FMELVNGFMY AFCFFAISQM YAYFENINFY ITSNFRFLDR YYGVFNKYFI

1251 NYAIIKLKEI TSDLLIKYER EAYLSMKKYG YLGEVIAARL SPKDKIMNYV

1301 HETNEDIMSN LRRYDMENAF KNKMSTYVDD FAFFDDCGKN EQFLNERCDY

1351 CPVIEEVEET QLFTTTGDKN TNKTTEIKKQ TSTYIDTEKM NEADSADSDD

1401 EKDSDTPDDE LMISRFH

**-Cytoadherence linked asexual protein [*Plasmodium falciparum* 3D7], CLAG3.2, PF3D7_0302200**

1 MVSFFKTPII IFFFLLCLNE KVLCSINENE NLGENKNENA NVNTPENLNK

51 LLNEYDNIEQ LKSMIGNDEL HKNLTILEKL ILESLEKDKL KYPLLKQGTE

101 QLIDISKFNK KNITDADDET YIIPTVQSSF HDIVKYEHLI KEQSIEIYNS

151 DISDKIKKKI FIVRTLKTIK LMLIPLNSYK QNNDLKSALE ELNNVFTNKE

201 AQKESSPIGD HGTFFRKLLT HVRTIKENED IENKGETLIL GDNKIDVMNS

251 NDFFFTTNSN VKFMENLDDI TNQYGLGLIN HLGPHLIALG HFVVLKLALK

301 NYKNYFEAKN IKFFSWQKIL EFSMSDRFKV LDMMCNHESV YYSEKKRRKT

351 YLKVDRSSTS MECNILEYLL HYFNKYQLEI IKTTQDTDFD LHGMMEHKYI

401 KDYFFSFMCN DPKECIIYHT NQFKKEANEE NTFPEQEEPN RQISAFNLYL

451 NYYYFMKRYS SYGTKKTLYV HLLNLTGLLN HDTRAYVTSL YLPGYYNAVE

501 MSFTDDKEFS TLFESLIQCI EKCHSDQARQ ISKDSNLLNN ITKCDLCKGA

551 FLYANMKFDE VPSMLQKFYV YLTKGLKIQK VSSLIKTLDI YQDYSNFLSH

601 DINWYTFLFL FRLTSFKEIA NKNVAEAMYL NIKDEDTFNK TIVTNYWYPS

651 PIKKYYTLYV RKHIPNNLVD ELEKLMKSGT LEKMKKSLTF LVHVNSFLQL

701 DFFHQLNEPP LGLPRSYPLS LVLEHKFKEW MDSSPAGFYF SNYQNPYIRK

751 DLHDKVLSQK FEPPKMNQWN KVLKSLIECA YDMYFEQRHV KNLYKYHNIY

801 NINNKLMLMR DSIDLYKNNF DDVLFFADIF NMRKYMTATP VYKKVKDRVY

851 HTLHSITGNS VNFYKYGIIY GFKVNKEILK EVVDELYSIY NFNTDIFTDT

901 SFLQTVYLLF RRIEETYRTQ RRDDKISVNN VFFMNVANNY SKLNKEEREI

951 EIHNSMASRY YAKTMFAAFQ MLFSTMLSNN VDNLDKAYGL SENIQVATST

1001 SAFLTFAYVY NGSIMDSVTN SLLPPYAKKP ITQLKYGKTF VFSNYFMLAS

1051 KMYDMLNYKN LSLLCEYQAV ASANFYSAKK VGQFLGRKFL PITTYFLVMR

1101 ISWTHAFTTG QHLICAFDPK RCTPDCKNST SYKSPQSFFY GWPPSSETYL

1151 FFYFFTNLYL DAYKSFPGGF GPAIKEQTQH VQEQTYERKP SVHSFNRNFF

1201 MELVNGFMYA FCFFAISQMY AYFENINFYI TSNFRFLDRY YGVFNKYFIN

1251 YAIIKLKEIT SDLLIKYERE AYLSMKKYGY LGEVIAARLS PKDKIMNYVH

1301 ETNEDIMSNL RRYDMENAFK NKMVTYVDDF AFFDDCGKNE QFLNERCDYC

1351 PVIEEVEETQ LFTTTGDKNT NETTEIKKQT STYIDTEKMN EADSADSDDE

1401 KDFDTPDNEL MIARFH

**- Cytoadherence linked asexual protein; CLAG9 [*Plasmodium falciparum* 3D7], PF3D7_0935800**

1 MIIWFIQPTI FYIIFILARN IQCTYKGDNI NEIKSILDND ELYNSLSNLE

51 NLLLQTLEQD ELKIPIMKGD LDKYLNMSNF KILNELNADG AEKPYIIPTS

101 NCSANDIVKY EHTLKTQITL EYKPEISDML KRKNIVVRTL KIIKFMQTPM

151 SAYKNTNNIK QSLLEMNKLF TNKEKKLNEH TINALRLRDR IFNTNNLTHR

201 SIKKGISTYM PIDTKSDIID YDDLLFTNHP SIELMENLDK LANYYHIGIF

251 NMIGSHYIAV GHFITLKLAL KNYKKYFEIG SLKYLNWQSI LKFNQSDRFK

301 VLDLICDESS WYEGQMKRRE QYLKNNIFST SEECSVLEFL IHHMNKYQME

351 LYSKMHKLSL NVQIYLENKH LKEKFLQFMC RSRKECNIYE SDRFKQEQEK

401 GIEFHDNNNY KFSQENDPVS KVVDPFNLFT NYFYFIKYYS VFNSDHIIYM

451 HLLNFVGVLN GNNNAYVSSL YLPGYYNAIQ LSYKDQVGLK ELYQNLVKCV

501 EKCYIRNRKN RSFSHRIASI FRHKKFDSSK CSICEGTLLY INDQSQDKMS

551 MAQKFYIFVT KILKVNNISS FITNMNIYED YSNYLMHDLN WYTFLFLFRM

601 TTYKDIPNYS ISNAMYLNIK DEDDTKRTMV TFQWMPSTIK RMHNYRIRKY

651 ISIYLLEELE KLIDNKLIEK LKKCITFLIH LNAFLQLDFF SYLNETPANL

701 QHPFPISMMI EARFKDWFIQ YLTGFFFINY DDANTRYNMP ENMKRGTFIP

751 PKYSKWNIHL KRFIDEAFLM YFNQKHALTL FKYHNPYNIS NKIMLMRDTF

801 ELYTKNYDQL IFGADIMLLR KTFSCTPMST KVWDRVKYYL HNIIGNPINF

851 YKHGLIYAYT LNKAMLKEVV NDFFVIYKMN KDLFSETSFL QTVYLLFKKI

901 QGTYFSHRRN DDVSMNNIFM FNVEKNYSKM SQADREKEIH ESMASRFFAK

951 NLFTVFQMMF VIQISNDVDK LDRIYGKADM LRLSVHDEPF LRFAYAYYGS

1001 MYDKLTNVFF PMNIKKPTIQ LKYGKTFIMA NLYYLCSVLF SMYNLNNLGL

1051 LCEYQAIGSA NFHSYKKMSQ FIDKKFIPLV FYTLKARTEG IIGKEWYKMV

1101 FNDFDGKSMA NTWPYFGYYM GGNMLYRNIL YFPNHLPEEL RKQTKGVELQ

1151 QPEYEPSVHS IDWQVGYAIS HGLSLSFFTF GMMKAYAYFE NVIFFLRNSI

1201 RIFDRFYSIL ENYVCMYIKR LFNKLTVDKL LKAMSRAYTS TKKEGAYEEA

1251 MVSRVRNKEN VVQEVQEDKG TDITPLPTFD IMDSKQNTNY MYNDNEDYFD

1301 DLDDNEQFLN SKDLLYYDDG IDRTKRYELI PLQRYRYDPF

**4. 1G5 mAb:**

**- Rhoptry associated membrane antigen [*Plasmodium falciparum* 3D7], RAMA, PF3D7_0707300**

1 MNVLLLSLLV VQNIVTYLEQ IKNGISGHYT EDHNIKNNNC ISFSDYERSI

51 KNFSISSHAE NNYDNIINEY KKIKDINNNI NILSSVHRKG RILYDSFLEI

101 NKLENDKKEK HEKEDEYEDN DESFLETEEY EDNEDEKYNK DEDDYAESFI

151 ETDEYEDNED DKYNKDEDDY SESFIETDEY DDNEEEQYNK DEDDYADSFI

201 ETDHYENNDD KNEEEEEYND QDNDYGYNFL ETDEYDDSEE YDYDDKEYGE

251 SFLEKEEGEE MKDEEMKDEE MEDVEMKDEE MKDEEMKYDE MKNEEMKYDE

301 MKDEVMKDEE MKDEQMKYEE FKNEEFKNEE FKNEESKNEE SKNEESKNEE

351 SKNEEFKNEE SKNEEFKNEE FKNEDMSYDE YMGYKKKEED ESYNTFNGTK

401 KNNTSNSFLE KDLQGDSDDE LHSTFYSKNV DKENYDDKNI FYGYSDNDDE

451 SFLETDSYEE YEDEDKDVED EYEESFLQND EKKMVFYDLY KPEENESYYE

501 KKQKKEEKEE KEEKEQSLNK QNDMEDQEDN EEYKFEEENK EDLLDVQQDE

551 ELPSEGKQKV KGKSFDNEHL NEIQNVSDVH AFIQKDMKYL DDLIDEEQTI

601 KDAVKKSAYK GNKKLGNNKK SQIILEEEPD ENFEEDADEE LNKLMEQEKN

651 IVDKEIKNSK ANKSNKKLQF NNTNKQNKMY MKNEYNNKTK NNKNNKFEQQ

701 NYDESYMDDD YEQNEEFNDN NQSEDMKETN ELDKINDELL TDQGPNEDTL

751 LENNNKIFDN KFVAHKKREK SISPHSYQKV STKVQNKENM ENKEKKQLIN

801 DEAAMTAEEL VELENTEDVN TPTMVETEEI DFDENGNKSS SSISYISSIV

851 FLMVTLLYFM N

**- Rhoptry neck protein 3 [*Plasmodium falciparum* 3D7], RON3, PF3D7_1252100**

1 MNKYWLYIAY VYLLLNILSK CNKIENNNNK VKNLTVYNND IGQYFKKKDI

51 QCKHHIEINQ DSNHNEYSFL SLKASSIFNQ YYVAKLINTL LYRGFHLNVY

101 FHKNVAMYES FSRTNFFFYL TILNKKNVKK IVKIISKAER SSNKRKFNVF

151 KKYLISEFDY PNFEIDDTVK NEMNQAILVY KKAKSDSYWS VMDALKKDGL

201 LLARTFLSVS FVQSLRGIIG LINHKLIDLC FTNAYVFNHV ASFDKLIMNN

251 IFGVIMSYVF KSLLLFFYPL IIPFRGAFAF AISAFCITQL GKIVFAIYKN

301 LRQLYRISYR KIYSIVLKVK LRNEPELKKY AMKLLYGDAL IMITKIWKLS

351 YVNVSEHLNG KNVYPILNNL FEKNLGTGFF DFSNSLFKYV MNYLEEINLL

401 NAKSVDVEKE LILNRHNFKL LLKILKITKK SLLYEESYMK ISVANLLTKF

451 YTLILVNIEH ISKLNPKEHF YNDLDNEFKH IYQDQMFELF IQKISSDIVR

501 KPFIKRNIGR IDKGSIELTL ALIKVKLLHY KPTLNNPYSS LYFDENLKKQ

551 LNYAFKLIII GSTSIIASLA NYGERYGVLK QCPLDIVKNL NQQCEYVSFE

601 IKKLIFPINI FVNLLIPLFL PYDDVLVDKN IIDEVKKFFN VIIDTDDSYL

651 QKYMKTTINT IRKSKDLDIN NVNYEEEMEK IVVQEAHKVI EEINKERRAS

701 FKFQDFVVKE DSVVLYNKSG IQYSFKFDHL NRKEDFMRII GSYQFKNPIG

751 YQTSQLVFEP QNNHIGSLMV GSNKNTFDGK LSVCHMCYKE DDDSLVVDAI

801 FSILWSSGID RFSAFIFASF IGAVKQTYHQ GTSWKRALSN MAPTEFYEMH

851 KIFMDKSVYG KEKSQNYFLK NIRKYRFQFS RGSFSRMFRT FLENSLNKIN

901 FFNSEEAIII LVMSTLYALY KNIEKFDIPL KETYKLYQQK LIESYYHVDK

951 YSHHYETYTL NIRRKKYNAL VKSEHEKAQE ASQGEGKDEA ENAELKKRVE

1001 KRINFNDIQL TMEDEVVRNT KYLQLELQAK PQEREKIIQY LTYKYKEIPN

1051 HEMFPHLPHQ CYFLLYYNYE PFLEKNLHGL EGVVSKITRK SVLSRYLKNI

1101 NLIQPEKTRF ASIKLKDLIN ILCGTHMFLK KEHITFDEIY KYENSNDALK

1151 HLLIIVALLR IEKRTNRYSW TTYLTLQKQL DERKRYYDTP FKYLFLKISK

1201 VRRFYGSAKK KMLLGFGKKF KGVRFLNILR YTRFFEIMEY AESINEFYPY

1251 CYIKFEDVLT FSNVFYKFKY SLLRYTTSKQ SVRNVVNNFN LSPQTTGNNE

1301 NLLLRIYRFI ELIIKKKYNV DIQHVRKDDK DFNRHAYNKN EYILNDIKFN

1351 PNIEQYLRQL TGFIKLLTDM KFTNFLFLRN LYYFVKFYAV TGDLTYSINN

1401 SSIYLCSRNY LEFILNSIIE FENFKKFMVK IKEKFDIEKY PIDPYNFQTE

1451 CYSIDSVKTY RFFLSDLDKL KSYQDIENLQ NVSSFYKDYL YMGLFYENLD

1501 VRNLNVYYNF NKKKNQDHKA HGSNVFLDGG LSLSKNMSFS DINDLSESIQ

1551 NYLYLEKFLA DSNIPSIPYQ GFSVRRVNDG MHVTYNNRST TPPLYKDNVL

1601 DQFELVGKSL ISEYFQKVKC SFIQYPFNLY YWLVLNPGKP NISLSSKTGL

1651 IKEDDLIPKT VEEKLKEEEE HDIKIVENIL SEDIFDFKDK SDDDQSTTAD

1701 VLSTDTDDSE TDAEKTSNNS NTLHKKETPA MKYNMNIAQD EINRQNDVSK

1751 NTTYAENNEY TSENITKPSD QNTENYGTKM PSPSFIQIDK VNQQDQQNDA

1801 HIESSKNTQE NLNYDDNTNM YEENKNDKKN KKLNNNTKEI SFLERRETNP

1851 IPFSSNMINE KNKEAHKEHY GNFNRPYNIY VPKKDIFRNF HLVVKVVHAL

1901 ISLNKFSMVS PDTILKKGIE SMKKKHSLKM IKNINPFIHK MILDMNETIQ

1951 KLKSDSEKAY GGPKADIISL YKIVEFQLFN QYIIYPPLKR LTKKELKYIL

2001 DVVNQGYYFY LKNVITKLKR ENLQKSKVVK IFNNFQEFSN LLDDDGVDKL

2051 YNIFTETFKC KNIKCFDLLF QGFLTEQYNN IVYRYTHDHD AMNSYNDNIF

2101 NNKELLNKIF RRAYDNYFIT PVKNNQPKKL ISLEKPSLEQ PVLTLEEFAY

2151 GFHEFGNKIE KWRSMLTLFN IRHIYINICH MLLSVKHSIQ RSHRVFMHKF

2201 GFFGIFRRKR EIYIP
